# Meeting people where they are: implementing hospital-based substance use harm reduction

**DOI:** 10.1186/s12954-022-00594-9

**Published:** 2022-02-09

**Authors:** Rachel Perera, Louise Stephan, Ayesha Appa, Ro Giuliano, Robert Hoffman, Paula Lum, Marlene Martin

**Affiliations:** 1 San Francisco General Hospital, San Francisco, CA USA; 2 Department of Medicine, University of California San Francisco, 1001 Potrero Avenue, San Francisco, CA 94110 USA; 3 Frank H Netter MD School of Medicine, Quinnipiac University, North Haven, CT USA; 4 Division of HIV, Infectious Diseases and Global Medicine, University of California San Francisco, San Francisco, CA USA; 5 San Francisco AIDS Foundation, San Francisco, CA USA; 6 Drug User Community Health, San Francisco, CA USA

**Keywords:** Harm reduction, Hospitals, Addiction, Safer use supplies, Community engagement, Health systems

## Abstract

**Background:**

Hospital-based addiction care focuses on assessing and diagnosing substance use disorders, managing withdrawal, and initiating medications for addiction treatment. Hospital harm reduction is generally limited to prescribing naloxone. Hospitals can better serve individuals with substance use disorders by incorporating harm reduction education and equipment provision as essential addiction care. We describe the implementation of a hospital intervention that provides harm reduction education and equipment (e.g., syringes, pipes, and fentanyl test strips) to patients via an addiction consult team in an urban, safety-net hospital.

**Methods:**

We performed a needs assessment to determine patient harm reduction needs. We partnered with a community-based organization who provided us harm reduction equipment and training. We engaged executive, regulatory, and nursing leadership to obtain support. After ensuring regulatory compliance, training our team, and developing a workflow, we implemented this harm reduction program that provides education and equipment to individuals whose substance use goals do not include abstinence.

**Results:**

During a 12-month period we provided 195 individuals harm reduction kits.

**Conclusions:**

This intervention allowed us to advance hospital-based addiction care, better educate and engage patients, staff, and clinicians, and reduce stigma. By establishing a community harm reduction partner, obtaining support from hospital leadership, and incorporating feedback from staff, clinicians, and patients, we successfully implemented harm reduction education and equipment provision in a hospital setting as part of evidence-based addiction care.

*Trial registration*: Commentary, none.

## Background

Unhealthy substance use and substance-related deaths are rising at astounding rates. In the 12-month period ending May 2021, over 100,000 individuals in the US died of drug-related overdoses [1]. Substance use disorder (SUD)-related emergency department visits and hospitalizations have simultaneously increased [2].

Hospitalized patients with SUD have longer lengths of stay and higher self-discharge and readmission rates than those without SUD, often related to inadequately treated withdrawal, pain, and stigma [3, 4]. Hospital-based addiction care is focused on diagnosing SUD, treating withdrawal, initiating medications for SUD, and prescribing naloxone for overdose reversal [5, 6]. However, these interventions alone fail to meet the needs of individuals who will continue to use substances.

Hospitals can better serve patients with SUD by more fully integrating harm reduction. We describe the implementation of harm reduction education and equipment provision (e.g., syringes, pipes, fentanyl test strips, and other safe use supplies) in an urban, safety-net hospital. To our knowledge, this is the first published guideline of how to integrate harm reduction education and equipment distribution in a US hospital as part of evidence-based addiction care.

## Harm reduction

The National Harm Reduction Coalition defines harm reduction as “a set of practical strategies and ideas aimed at reducing negative consequences associated with drug use” [7]. Harm reduction recognizes that harms from substance use are real and that people use substances for complex reasons including racism, poverty, trauma, pain, homelessness, and gender-based violence [7]. Harm reduction can be practiced across substances and route of use. Examples of harm reduction include supervised consumption sites, managed alcohol programs, sobering centers, safe supply, and syringe service programs (SSPs).

Our hospital-based harm reduction intervention focused on education and equipment provision given patient needs and legalities. As our intervention is most similar to SSPs, we briefly review their history and evidence. People who use drugs opened the first SSP in Rotterdam in 1985 in an effort to reduce hepatitis B infections [8]. People who use drugs continued spearheading SSPs throughout the 1980s given newfound awareness of the prevalence of HIV among people who inject drugs [8]. Today, SSPs distribute substance use equipment and often provide supportive services including hepatitis C and HIV testing and treatment, overdose prevention, naloxone, condoms, and addiction service referrals. SSP interventions reduce viral and bacterial infections and increase SUD treatment engagement [9, 10]. Moreover, people who engage in SSPs are five times likelier to enter addiction treatment and three times likelier to abstain from substances that those who do not [11].

As of August 2019, 31 US states and the District of Columbia have authorized SSPs, though regulations vary geographically [12]. In California, where we are based, Health and Safety Code 11364.7(a) allows for distribution of syringes and other materials deemed by the local or state health department to prevent infection transmission, drug overdose, injury, and disability by a public entity, its agents, and employees through a certified SSP [13]. It is unclear whether healthcare sites are considered SSP extensions as a “public entity.” In addition, the same safety code criminalizes possession of safer use supplies––except for needles obtained through SSPs.

Given complex regulations and legal concerns, most US healthcare settings have not implemented harm reduction equipment provision. However, in Canada, where regulations are different, evidence shows hospital-based harm reduction interventions improve patient–clinician experiences, reduce stigma, reach populations missed by traditional interventions, and advance health knowledge [14–16].

## Harm reduction implementation

### Our setting

The Addiction Care Team (ACT) is an interprofessional consultation service composed of patient navigators, licensed vocational nurses, and clinicians that provide addiction services to emergency department and hospitalized patients in an urban, safety-net hospital [4]. We tailor care to patients’ circumstances and goals with a focus on evidence-based addiction treatment, harm reduction, and linkage to community services.

With the aims of (1) integrating harm reduction as an evidence-based SUD service; (2) engaging patients in SUD care regardless of their stage of change; and (3) reducing stigma toward people with SUD, ACT implemented harm reduction education and equipment provision during hospitalization.

### Identifying harm reduction need and a community partner

In May 2020, ACT navigators assessed the harm reduction needs of 30 hospitalized patients whose substance use goal was not abstinence. Patients identified the harm reduction supplies that could help them use substances more safely and whether they wanted to receive supplies at hospital discharge.

Our team also contacted the San Francisco AIDS Foundation, a community-based organization that serves people who use drugs and people living with HIV. The organization agreed to supply harm reduction equipment. The needs assessment and San Francisco AIDS Foundation education allowed our team to center lived-experience in local substance use practices, supply trends, and harm reduction strategies, which served as the basis for our intervention.

### Obtaining executive and nursing leadership support

We contacted the hospital’s executive leadership (CEO, CMO, CNO) and the Director of Regulatory Affairs and described our goals and needs assessment results, which demonstrated patient need for harm reduction supplies at discharge. After confirming that a community partner would provide harm reduction equipment and establishing a process for documentation, leadership approved this intervention. This process was straightforward since we were not purchasing harm reduction equipment.

Our team also met with nursing leadership. Engaging nurses was critical given their patient facing role and likelihood of observing harm reduction equipment distribution. Nurses expressed concerns about the workflow, regulations, safety, and in-hospital substance use. To address these, we obtained nursing input in operationalization, reviewed harm reduction evidence, and acknowledged the strain that substance use can have on patient, staff, and clinician relationships. We also discussed how harm reduction could alleviate friction, foster alliance, increase treatment engagement, and reduce stigma [14, 17].

### Operationalizing a workflow

ACT members attended trainings on harm reduction principles, safer injection practices, and overdose prevention [7]. One navigator had worked at a SSP and reviewed substance use equipment with our team. Another navigator compiled harm reduction resources into a shared directory. Our community partner also recorded a peer navigator led harm reduction equipment training that we use to onboard new members.

We assemble harm reduction kits by substance and route of use to streamline distribution, and adjust each kit based on individual patient needs. All kits include information about community harm reduction programs, mental health services, and overdose prevention. See Table 1 for harm reduction kit components by substance and route of use, and rationale.

**Table 1:** Harm reduction supplies and education by substance and route of use and rationale

Substance and route of use (when applicable)	Supply or education	Harm reduction rationale
Alcohol	“Rethinking Drinking” brochure from the National Institute on Alcohol Abuse and Alcoholism on safer drinking ^a^. We also provide tips to reduce the harms of alcohol use including: -Decrease drinking days and drinks per day -Eat before drinking -Alternate water with alcohol or dilute alcohol -Create a drinking tracker card -Make a safer drinking plan (e.g., carry condoms and lock car keys) -Naltrexone and other medication education	These interventions may improve individual health measures, such as cirrhosis progression and alcohol withdrawal severity. They may also reduce risky physical and social behaviors, psychiatric hospitalizations, and symptoms of depression and anxiety, and improve self-efficacy, social functioning, and workplace productivity^﻿b^.
	Sobering center flyer	Informs patients of a location with medical staff where they can stay while intoxicated.
	Medically supervised withdrawal management facility flyer	Informs patients about a medically managed alcohol withdrawal facility where they can also link to residential treatment.
	Food assistance resources	Access to a nutritious diet is important as reduced dietary intake and changes in nutrient absorption due to alcohol use may mediate long term health impacts.
Stimulants	Safer stimulant use education	We counsel patients to prepare for a decreased awareness of the need to eat, sleep, and drink, increased libido, and a higher likelihood of sleeplessness and psychosis. Tips include drinking water, eating, resting, carrying condoms, and using in a safe space with a trusted individual. Many patients do not realize stimulants carry overdose potential. We discuss overdose risk and safer use strategies, as outlined in this table depending on the route of use.
Tobacco	Toothpicks and gum	Oral fixation to reduce cravings and reduce frequency of use.
	Education	We discuss the health benefits of stopping tobacco use, as well as resources for financial aid for nicotine replacement therapy through 1-800-No-Butts.
Opioids	Safer opioid use education	We review overdose risk and safer use strategies, as outlined in this table depending on route of use.
Smoking opioids and stimulants	Pyrex pipe	Reduce need to share equipment or use broken equipment. Reduce risk of cuts and burns, and subsequent infection transmission by providing a pipe that does not overheat and a barrier to directly touching the glass. Educate that smoking increases the risk of cardiovascular and respiratory harms. Educate that smoking carries reduced overdose and infection risk compared to injecting^c^.
	Rubber pipe mouthpiece	
	Steel wool	Cleans cocaine pipe residue to avoid re-inhaling prior substance.
Smoking fentanyl	Clean foil	Reduces need to reuse materials and can help encourage people to switch from injecting to smoking, which reduces risk of infection and overdose^c^.
	Pyrex pipe and rubber mouthpiece	See "smoking opioids and stimulants" section for pyrex pipe and rubber mouthpiece information.
	Education	We review overdose prevention, infection risk reduction, and other safer use strategies.
Inhaling opioids or stimulants	Clean straws	Reduce the need to reuse and share materials, thus reducing infection risk.
	Education	Counsel patients to adequately crush substances to reduce injury to nasal mucosa. We share that smoking carries lower overdose risk and infection risk than injecting^c^.
Injecting opioids or stimulants	Sharps container	Reduces presence of used needles in community by providing a disposal method.
	Needles	Reduce risk of viral and bacterial infections by reducing sharing and reuse.
	Cooker	Reduces risk of infections.
	Tourniquet	Reduces need for multiple injections by making veins more accessible.
	Cotton pellets	Filter out large particles from drug solution and reduce reuse of pellets.
	Alcohol wipes	Clean skin to reduce infections.
	Vitamin C	Used to change cocaine from free base to water soluble, acid salt form for injecting cocaine. Patients may otherwise use citrus fruits like lemons, which carry infection risk.
	Education	Injecting is associated with highest risk of infections and overdose^c^. Thus, we educate about: -Cleaning skin -Rotating injection sites -Reducing punctures by using a tourniquet and heat to find veins and injecting with bevel up to reduce infections and preserve veins -Injecting in safer anatomic sites (e.g., avoiding groin and neck vessels)
All kits may include	Fentanyl test strips and education	Fentanyl test strips reduce unintentional ingestion of fentanyl and overdose risk^d^. We discuss that fentanyl strips cannot detect all fentanyl analogs and should be used in addition to other precautions (e.g., carrying naloxone, using test doses, not using alone). We also discuss that fentanyl test strips are not recommended for amphetamine testing due to high false-positive rates. Fentanyl test strips can be used for pressed pills, heroin, and cocaine.
	Condoms	Riskier sex is more common while using substances. Condoms can prevent sexually transmitted infections.
	Never use alone flyer and education	Never use alone is a confidential and anonymous overdose prevention line (1–800-484–3731). The operator asks for a first name, location, and the phone number the person is calling from. The operator stays on the line while a person uses and calls 911 if the person stops responding. Using alone increases overdose risk.
	Naloxone	Opens discussion that opioid overdose and death are possible outcomes of substance use. Reverses opioid overdose.
	Overdose prevention education	Allows discussion of overdose risk and includes tips such as not using alone, using a test dose, using through a safer route (e.g., inhaling rather than injecting), and carrying naloxone.
	Local resources	Includes overview of local SSPs and other services to encourage and enable patients to connect to outpatient resources.
	Additional resources	Information about rectal substance use and wound care as well as widely available resources from the National Harm Reduction Coalition^c^ and NEXT Distro.^e^

^a^Rethinking Drinking. National Institutes of Health: National Institute on Alcohol Abuse and Alcoholism. U.S. Department of Health and Human Services. https://www.rethinkingdrinking.niaaa.nih.gov. Accessed 14 Aug 2021

^b^Charlet K, Heinz A. Harm reduction: a systematic review on effects of alcohol reduction on physical and mental symptoms. *Addict Biol.* 2017;22(5):1119–1159

^c^National Harm Reduction Coalition. https://harmreduction.org. Accessed 15 July 2021

^d^Krieger MS, Yedinak JL, Buxton JA, et al. High willingness to use rapid fentanyl test strips among young adults who use drugs. *Harm Reduct J.* 2018;15(1):1–9

^e^NEXT Distro. https://nextdistro.org/. Accessed 20 Jan 2022

We developed harm reduction hospital workflow with patient, staff, and clinician feedback. We give kits at discharge to reduce the risk of misunderstandings between staff, clinicians, and patients. We inform the patient’s care team when we give a harm reduction kit and invite them to participate in education. Our documentation includes the equipment provided, as recommended by our hospital’s regulatory department.

### Piloting harm reduction education and kits

In August 2020, we piloted our intervention on two medical-surgical floors to test and adjust the workflow. During this time, staff and clinicians alerted our team that many remained unaware of this intervention, highlighting the need for education. We also missed distributing kits to patients discharged when ACT members were unavailable. Thus, we updated our workflow to review kits with patients upon consult and store them in a locked cabinet in the patient room to be opened by nurses at discharge rather than attempting discharge delivery. This reduced the number of patients who did not receive a kit and allowed access regardless of ACT presence at discharge. In January 2021, after revising our workflow based on the pilot, we expanded it hospital wide.

## Results

From August 2020 to July 2021, we provided 195 harm reduction kits. As we focused on implementation, we did not collect detailed data. However, among 57 individuals served by this intervention we found that kits were given across substances (opioid, alcohol, cocaine, methamphetamine, benzodiazepine, tobacco, and cannabis), race/ethnicity (Latinx 29.9%, white 28.1%, Black 26.3%, Asian 8.8%, other 6.9%), and housing status (experiencing homelessness 38.6%, marginal housing 28.1%, housed 31.6%, unknown 1.7%).

## Discussion

We successfully implemented this intervention by assessing the harm reduction needs of patients with SUD, community partnership, obtaining hospital, regulatory, and nursing leadership support, training staff, and developing a workflow. This intervention educated and engaged patients, staff, and clinicians and reduced stigma.

### Education and engagement

Patients, staff, and clinicians reported being unaware of infection risks associated with smoking and inhaling substances and reusing or sharing cookers. They were also unaware that stimulants warrant overdose prevention and that harm reduction for stimulants includes naloxone, fentanyl test strips for cocaine, test doses, and a 24-h overdose prevention hotline if using substances alone. Clinicians began consulting ACT for harm reduction support after this intervention launched, suggesting increased harm reduction awareness.

Patients new to San Francisco reported reusing and sharing equipment. They were relieved to receive equipment and learn about local harm reduction programs during hospitalization. Patients who smoked or inhaled substances appreciated learning that SSPs offer pipes, pipe covers, and fentanyl test strips. Those with limited English proficiency were generally unaware of naloxone. Patients with limited mobility and those discharging to a location without harm reduction programs were thankful for discharge access to harm reduction kits.

Patients who initially did not want ACT services became interested upon learning we offered harm reduction equipment. Patients commented that our harm reduction services allowed for more open discuss about substance use goals. Multiple patients contacted us when they were discharging before their expected date to ensure they received a harm reduction kit.

### Stigma

Patients, staff, and clinicians reported that this intervention reduced addiction-related stigma. Staff and clinicians expressed legality concerns and worried providing harm reduction equipment led to increased substance use. Some nurses expressed discomfort when asked to give harm reduction supplies at discharge. We approach these moments as opportunities to describe harm reduction evidence, reflect on how we can improve addiction care, and discuss the role of stigma.

Many patients shared previous experiences of discrimination, which had delayed and prevented healthcare engagement. Several patients contrasted those instances to their current hospitalization. They expressed relief about discussing substance use and their needs and goals without judgment during hospitalization. Patients endorsed excitement about improving personal well-being and the well-being of loved ones with harm reduction. Only one patient expressed that offering needles made him feel triggered to use drugs.

### Challenges

Currently, our intervention relies on ACT seeing a patient. Since ACT demand exceeds capacity, we must increase access to harm reduction education and equipment regardless of ACT availability. While we aim to educate staff and clinicians, we are limited by clinical demands, and there is a need for more systems-wide addiction education to staff and clinicians.

## Conclusion

Incorporating harm reduction in healthcare systems meets people with SUD where they are and is an essential evidence-base addiction service. This intervention allowed us to advance evidence-based care for people with SUD, better educate and engage patients, staff, and clinicians, and reduce stigma.

Our findings are limited given informal feedback from patients, staff, and clinicians. We plan to evaluate how this intervention affected patient outcomes and patient, staff, and clinician experiences. We need formal studies to build evidence for hospital-based harm reduction interventions so that they can be implemented more widely in the US.

While our hospital-based harm reduction education and equipment provision intervention is promising, we recognize that we must continue advancing addiction interventions. In hospital settings, this means expanding access to evidence-based addiction services. Further work to implement and evaluate more expansive harm reduction interventions, including supervised consumption sites and safe supply, is also necessary. Simultaneously, we must also address the factors that interplay with addiction including trauma, mental health, and housing access, and ensure equitable and low-threshold access to evidence-based addiction services for minoritized communities and those with limited English proficiency, who often face reduced access to treatment and worse addiction-related outcomes.

Harm reduction in hospitals is a critical component of evidence-based addiction care and includes, educates, and empowers patients. This guideline can help US hospitals incorporate harm reduction education and equipment provision.

## Data Availability

Not applicable.

## Abbreviations

SUD: Substance use disorder
SSP: Syringe service program
ACT: Addiction Care Team
